# Supplementary material for: Controlling astrocyte-mediated synaptic pruning signals for schizophrenia drug repurposing with deep graph networks
Source: PLoS Comput Biol. 2022 May 4;18(5):e1009531. doi: 10.1371/journal.pcbi.1009531 (PMC9109907; doi:10.1371/journal.pcbi.1009531)
Supplement: S1 Appendix — (PDF) [file pcbi.1009531.s001.pdf]

S1 Appendix

In this appendix we summarise the main characteristics of the employed Graph Convolutional Layers and Deep Graph Networks. GraphSAGE is a GCL that learns embeddings by sampling and aggregating features from a node's local neighborhood. Considering i the current node and k the current step, the new node representation $\mathbf{x}_i^k$ is computed as

$$\mathbf{x}_i^k = \sigma(\mathbf{W}^k \cdot (\mathbf{x}_i^{k-1} \,||\, \phi(\{\mathbf{x}_j^{k-1}, \forall j \in \mathcal{N}(i)\}))$$

where σ is a non-linear activation function, $||$ denotes concatenation, ϕ is an aggregation function, and $\mathbf{W}^k$ denotes the weight matrix. In our experiments, we used a mean aggregation function, so

$$\phi = \frac{1}{|\mathcal{N}(i)|} \sum_{j \in \mathcal{N}(i)} \mathbf{x}_j^{k-1}.$$

GAT is a GCL that leverages an attention mechanism to learn neighbors' influences. A GAT layer computes node representation as

$$\mathbf{x}_i^k = \sigma\left(\sum_{j \in \mathcal{N}(i) \,\cup\, \{i\}} \alpha_{i,j} \mathbf{W}_j^{k-1} \mathbf{x}_j^{k-1} \right)$$

where $\alpha_{i,j}$ is the normalized attention coefficient for node i with respect to neighbor j. Attention is computed as a single-layer MLP with LeakyReLU parameterized by the weight vector $\mathbf{a}$:

$$\alpha_{i,j} = \frac{\exp(\mathrm{LeakyReLU}(\mathbf{a}^T[\mathbf{W}^{k-1}\mathbf{x}_i^{k-1} || \mathbf{W}^{k-1}\mathbf{x}_j^{k-1}]))}{\sum_{l \in \mathcal{N}(i) \,\cup\, \{i\}} \exp(\mathrm{LeakyReLU}(\mathbf{a}^T[\mathbf{W}^{k-1}\mathbf{x}_i^{k-1} || \mathbf{W}^{k-1}\mathbf{x}_l^{k-1}]))}$$

where $\cdot^T$ means transposition.

ECC is a GCL commonly used in quantum chemistry prediction tasks [**?**]. It employs edge features in addition to the local neighborhoods to compute node representations. In such a scenario, the node representation is computed as

$$\mathbf{x}_i^k = \mathbf{x}_i^{k-1} + \sum_{j \in \mathcal{N}(i)} \mathrm{MLP}(\mathbf{e}_{ij}) \mathbf{x}_j^{k-1}$$

where $\mathbf{e}_{ij}$ is the feature vector of the chemical bond that connects atoms i and j.

Neural Graph Fingerprint is a DGN that can be seen as the neural equivalent of the ECFP. Differently from the static approach, it replaces discrete operations with their differentiable analogs. Furthermore, the hash function, whose goal is to combine neighbors information, is replaced with a single-layer MLP with a smooth activation function. The indexing function, which is used to combine into a single fingerprint vector the computed features, is replaced by a softmax function. Each node is updated by taking into consideration both atoms and bonds information from its neighborhood. Node representations are summed at each step to generate a graph-level representation, which are then summed together. More formally, the neural fingerprint of a compound, c, is computed as

$$\mathbf{h}_c = \sum_{k=1}^{r} \sum_{i=1}^{a} \mathrm{softmax}(\mathrm{MLP}^k \left(\sum_{j \in \mathcal{N}(i) \,\cup\, \{i\}} (\mathbf{e}_{ij} \,||\, \mathbf{x}_j) \right) \cdot \mathbf{W}^k)$$

where r is the radius of the fingerprint, a is the number of atoms in the compound, and $\mathbf{W}^k$ is the output weight matrix for radius k.
