# Supplementary material for: Controlling astrocyte-mediated synaptic pruning signals for schizophrenia drug repurposing with deep graph networks
Source: PLoS Comput Biol. 2022 May 4;18(5):e1009531. doi: 10.1371/journal.pcbi.1009531 (PMC9109907; doi:10.1371/journal.pcbi.1009531)
Supplement: S2 Appendix — (PDF) [file pcbi.1009531.s002.pdf]

S2 Appendix

Table 1. Hyper-parameter used for model selection when f_{out} = MLP.

		Configurations
ECFP	radius	2, 3, 4, 5
	length	2^7, 2^9, 2^{10}, 2^{11}, 2^{12}
optimizer	name	Adam, AdaMax, SGD
	lr	$1e^{-3}$, $5e^{-3}$, $5e^{-4}$, $5e^{-5}$
activation fun		ReLU, LeakReLU, Tanh
aggregation fun		mean, sum
batch size		512
epochs		900

Table 2. Hyper-parameter used for model selection when f_{out} = RF.

		Configurations
ECFP	radius	2, 3, 4, 5
	length	2^7, 2^9, 2^{10}, 2^{11}, 2^{12}
# trees		{30, 50, 100, 200, 300, 350, 400, 450, 500, 550, 600, 650, 700, 800, 4000, 8000, 16000}
tree depth		30, 40, 50, ∞
split quality metric		entropy, gini
min sample split		2, 4, 6, 10, 15, 20, 30, 35, 40
min sample leaf		1, 2, 3, 5, 6, 10
class weights		balanced

Table 3. The list of tested architecture configurations of f_{comp} and f_{out} for the models LinNN, MoNN, SAGENN, GaNN, ENN, NeFPNN.

	f_{comp} architectures	f_{out} architectures
LinNN	[200], [50], [50, 75]	[250, 150, 50, 3], [150, 50, 10, 3], [100, 50, 10, 3], [30, 10, 3], [50, 3], [5, 3], [3]
MoNN	—	[512, 256, 128, 64, 32, 16, 8, 3], [512, 128, 32, 8, 3], [512, 128, 32, 3], [512, 128, 3], [512, 64, 3], [256, 64, 3], [128, 64, 3], [512, 3], [256, 3], [64, 3], [3]
SAGENN	[512, 256, 128], [1024, 512], [256, 128], [128, 64], [1024], [512], [256], [128], [64]	[512, 128, 3], [256,128, 3], [128, 64, 3], [64, 32, 3], [32, 16, 3], [128, 3], [64, 3], [3]
GaNN	[512, 256, 128], [256, 128], [256, 64], [128, 64], [1024], [256], [128], [64]	[512, 128, 3], [64, 32, 3], [64, 3], [32, 3], [3]
ENN	[256, 128], [128, 64], [64, 32], [256], [128], [64], [32]	[128, 64, 3], [64, 16, 3], [32, 16, 3], [32, 3], [16, 3], [3]
NeFPNN	[128, 128, 128, 128], [128, 128, 128], [128, 128]	[256, 64, 3], [128, 64, 3], [256, 3], [128, 3], [64, 3] [3]
