# Supplementary material for: Controlling astrocyte-mediated synaptic pruning signals for schizophrenia drug repurposing with deep graph networks
Source: PLoS Comput Biol. 2022 May 4;18(5):e1009531. doi: 10.1371/journal.pcbi.1009531 (PMC9109907; doi:10.1371/journal.pcbi.1009531)
Supplement: Attachment — Submitted filename: Response to reviewers.pdf [file pcbi.1009531.s003.pdf]

The authors would like to thank the Editors and the Reviewers for their insightful comments. Please find in the following our rebuttal to the issues raised in the review, with reference to the major changes performed to the manuscript.

Reviewer #1:

(1) The authors developed a computational method for schizophrenia drug repurposing with Deep Graph Networks. The authors directly employed Deep Graph Networks to their problems but didn't propose novel methods.

We agree that the key contribution of the paper is not the introduction of a novel Deep Graph Network Model. Rather, our approach identifies compounds that reduce glial phagocytic activity for the treatment of schizophrenia, which, to the best of our knowledge, has not been proposed before. Thus, novelty of this work has to be sought into the DGN-enabled study of compounds that reduce glial phagocytosis rather than in the ML methodology. We are of course open to consider relevant works that we might have missed with respect to this specific topic, should the Review have knowledge of them.

(2) Do all compounds have the five concentrations, i.e., 1.39, 2.78, 5.56, 11.11, 22.22. If so, how can we make prediction if the compound has concentrations different from these?

We normalized the concentration by making the mean equal to 0 and standard deviation equal to 1, thus any continuous value can be used as concentration. Moreover, we provided the values of mean and std in the github repository.

(3) The authors introduce too much basic knowledge about the Deep Graph Networks and molecular fingerprint ECFP, and it is better to make them concise. The writing and organization could be improved for better readability.

As the reviewer suggested, we made the Model section more concise by moving a major part of the knowledge details into a separated section in the appendix (see S1 Appendix "Overview of the employed dynamic compound embedding modules based on DGNs").

(4)The source codes and datasets are not publicly available, and the web link provided by the author is empty. It is difficult to objectively evaluate the quality of the data and explore details about the model implementation.

The Github link is working now, and it contains data and implementation details. We apologize for not having provided such an access before.

Reviewer #2:

The AUROC of the top models (after sweeping hyperparameters) in validation on the held-out SPARK HTS data seems low (0.65-0.68), regardless of cross-validation approach and also poor in the risk-assessment test (Table 4—AUROC 0.64-0.69), compared to published performances of other LBVS models.

Our method identifies compounds that reduce glial phagocytic activity for the treatment of schizophrenia, which, to the best of our knowledge, has not been proposed before. Therefore, a fair comparison between our methodology and other models optimized to predict different

properties cannot be done. Of course we acknowledge the need for a baseline to assess the performance of our approach. For such a reason, we opted to employ the baseline ECFP + Random Forest (referred as MoRF in the article) as it is commonly used in literature to predict molecule properties [1-4].

[1] Lind AP, Anderson PC. *Predicting drug activity against cancer cells by random forest models based on minimal genomic information and chemical properties.* PLOS ONE. 2019.
[2] Zhao K, So HC. *Drug Repositioning for Schizophrenia and Depression/Anxiety Disorders: A Machine Learning Approach Leveraging Expression Data*. IEEE Journal of Biomedical and Health Informatics. 2019.
[3] Liu S, Alnammi M, Ericksen SS, Voter AF, Ananiev GE, Keck JL, et al. *Practical Model Selection for Prospective Virtual Screening.* Journal of Chemical Information and Modeling. 2019.
[4] Kapsiani S, Howlin BJ. *Random forest classification for predicting lifespan-extending chemical compounds*. Scientific Reports. 2021.

[...] Although the SPARK molecule set was examined in terms of structural diversity (based on Jaccard and Cosine distances of chemical fingerprints), it is possible that molecules of similar scaffold/chemotype were in spread across folds. This might be mitigated by clustering the training molecules and confining cluster IDs to specific folds. This ensures that predictions are made on chemotypes outside of the training set. Also, rather than showing the quartiles in Table 1, a plot of the distributions (as histograms) would be more informative—as well as some examples of molecule pairs at the different dissimilarity values. It is not obvious what the dissimilarity scores reflect—perhaps some statistics on scaffold diversity (Bemis-Murcko) would be useful to reader.
As the review suggested, we performed an analysis on the scaffold diversity in the dataset (which we have included in the section of the manuscript titled “Compound dissimilarity analysis”). We used the same methodology employed in the Compound dissimilarity analysis section. As it is clear from Table 1 there is a strong diversity between scaffolds. Moreover, Figure 1 shows that only few molecules in our data share the same scaffold. Indeed, there are 863 scaffolds associated with 1 molecule in the data.

Table 1: Cosine distance and Jaccard dissimilarity between molecules’ scaffolds in the dataset.

**Name**	**Avg±std**	**25 perc**	**50 perc**	**75 perc**	**Max**	**Min**
Cosine	0.8234±0.0750	0.7805	0.8091	0.8635	0.9999	0.7050
Jaccard	0.8990±0.0341	0.8838	0.9033	0.9182	0.9772	0.8243

Such results highlight that the scaffold distribution is long tailed. The compounds’ uniqueness in the dataset represents an index of strong complexity of the task and justifies why our predictive performance are in the range 0.64-0.69. Lastly, the plot of the scaffolds (Figure 2) does not

show any natural cluster of the fingerprint. In conclusion, it is unlikely that any other training strategy based on scaffolds could lead to better performance.

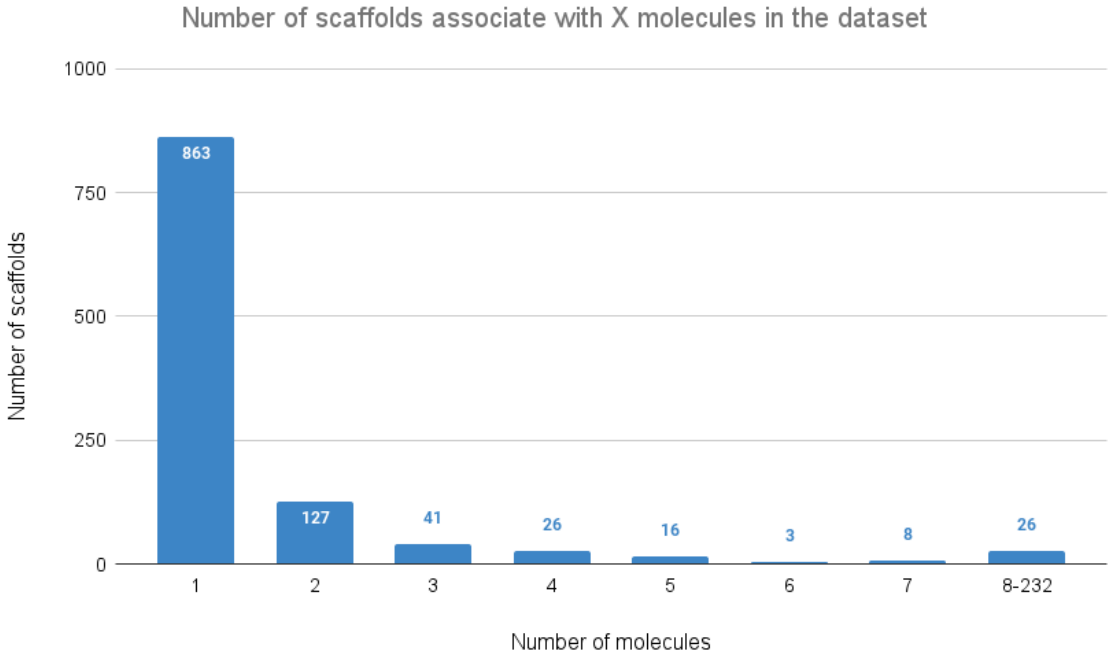

Figure 1

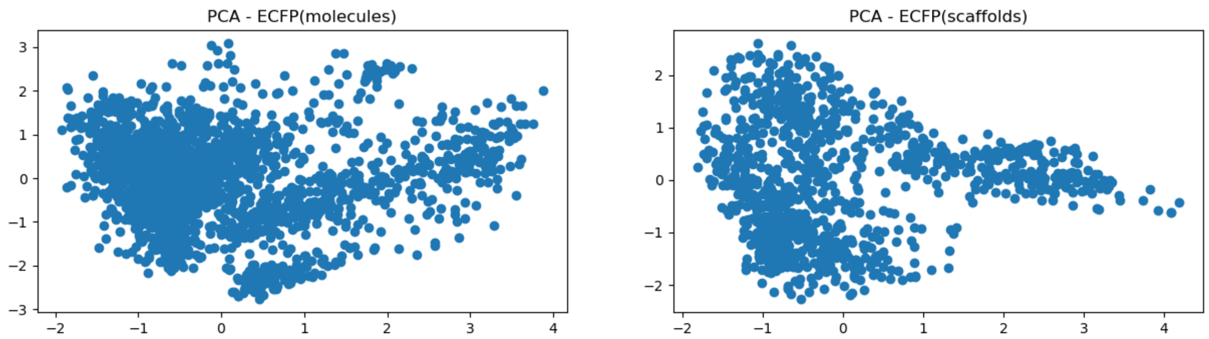

Figure 2

[...] the reviewer recommends a using just the compound representations as inputs and assigning activity labels based on the full dose-response curves (potency or binary/tertiary classes). This will reduce the number of instances from ~10000 to ~2000 but this would likely improve reliability of the labels and simplify the relationship between inputs and outputs. It's understood that some molecules showed both pro- and anti-phagocytotic activities depending on dose. Such complex responses exhibited by the compound instances could be a result of artifacts. Assay responses at the higher concentrations are probably less relevant and might merely reflect toxicity. Perhaps using the observed response (pro- or anti-phagocytotic) at the lowest concentration

point could resolve this issue. Also, since decreased phagocytotic activity is the therapeutically relevant endpoint, perhaps a simple binary classification could be used based on achieving some threshold decrease in phagocytotic response.

We thank the reviewer for the recommendation. We include in the manuscript a new section titled “Problem relaxation” containing new experiments considering a relaxed version of the problem. However, differently from the reviewer's suggestion, we believe that removing the concentration from the experiments will severely harm the benefits of our method, and undermine the clinical principles that inspired our study. Moreover, the choice of the concentration to keep is particularly difficult because the molecular activation has a sigmoidal shape. Hence, lower concentrations could lead to noise and artifacts, given that molecules can be inactive at such concentrations.

Higher concentration could also lead to less relevant results and might merely reflect toxicity, as the reviewer indicated. At the same time, picking a concentration at random in between high and low values does not seem a reasonable approach. Thus, we decided to simplify the task by keeping the concentrations and reducing the number of classes from 3 to 2, i.e., positive and negative impact on phagocytosis.

These findings of such experiments show that by relaxing the problem it is possible to achieve higher performance (~10 points higher than the original problem). Therefore, it is reasonable to assume these results as proof of the complexity of the original problem. Indeed, in this case the computational task of structure-function prediction is quite hard because phagocytosis is a multi-protein biological process. This suggests that multiple molecules could bind proteins in this pathway and that there might not be one ideal structure for influencing this process. Moreover, this situation, followed by the high dissimilarity between molecule structures, indicates that our method learns good compound representations and is not overfitting molecular substructures for making predictions.

The use of ATC codes and Chemical Abstracts topics terms to associate molecule target sites for assessing model predictive performance is interesting but not sufficient for demonstrating the utility of the model for VS on phenotypic endpoints. That the molecules prioritized by the model are frequently associated with CNS therapeutics does not demonstrate that the model is useful for predicting the anti-phagocytotic response. The work would be much stronger with follow-up testing on some or all of the 64 prioritized molecules from SWEETLEAD in the original SPARK assay. Are there other molecules (outside of SWEETLEAD) that have been tested in this assay (or similar assay) that could be evaluated by the model retrospectively? Are there other molecules shown to be active in published assay data (ChEMBL or PubChem BioAssay) on cell-based or biochemical targets related to synaptic phagocytotic activity? Perhaps these data could be used in retrospective test.

We agree with the reviewer that it will be very interesting to pursue follow-up tests on the prioritized molecules from SWEETLEAD. Unfortunately, we can’t recreate the assay and we can’t evaluate other molecules retrospectively. However, we want to point out that our purpose in that section was to repurpose a new library of compounds and propose a list of 64 good candidates. Then we analyzed how our candidates are usually employed. We apologize if our language was misleading and induced the false belief that we run a biological validation phase

to promote the goodness of the model. We believe that associated ATC codes and Chemical Abstracts topics terms can be a reasonable tool to show the utility of compounds. The fact that the literature analysis shows matching results with recent theory can be taught as a proof of the candidates' beneficial effect in our problem.
Lastly, we found evidence of the utility of some of our candidates (i.e., Loxapine, Dextromethorphan, Thioridazine, Trifluoperazine, and Cetirizine) in [1]. We observe that the experimental setting and the task is different from our's, because they developed a framework for drug repositioning by comparing transcriptomes imputed from GWAS data with drug-induced gene expression profiles. Thus, they are not performing structure-function prediction. We observe also that a complete match with our candidates is not possible because the authors report only the top-15 compounds. We included such findings in the "SWEETLEAD library repurposing" section.

[1] So HC, Chau CL, Chiu WT et al. *Analysis of genome-wide association data highlights candidates for drug repositioning in psychiatry.* Nature Neuroscience, 2017.

Table 1 – the degree units on the quartile values should be removed. Median (50%) is usually reported when reporting quantiles.
Thank you, we fixed the mistake.

SI Table 9 -- # trees range probably should go much higher – I see much larger forests used with ECFP input representations (n_estimators) in Liu et al., 2019. https://pubs.acs.org/doi/10.1021/acs.jcim.8b00363).
In our experiments we decided not to include larger forests due to the strong overfitting that is already present when using smaller forests. Moreover, the rationale of Liu et al. to employ such a large forest is that their datasets are 10 to 32 times bigger than our's. Nevertheless we run the simple CV with 4000, 8000 and 16000 trees as reported in Liu et al., 2019. As shown from Table 3 our original intuition was confirmed. Indeed the model achieves a similar validation score but higher training score with respect to smaller forests, suggesting that the model is overfitting. In this scenario, bigger forests do not improve the performance of the model. We included these configurations in Table 11.

**Table 3: Simple CV results of MoRF with number of trees equal to 4000, 8000, or 16000.**	
macro-AUROC score	TR: 0.9422 +/- 0.0003<br>VL: 0.6674 +/- 0.0079
Best configuration	*ECFP length:* 128, *ECFP radius:* 4, *criterion:* entropy, *max depth:* 20, *min samples leaf:* 1, *min samples split:* 10, *# trees:* 8000

Line 113 – Table 3 does not show that nearly all models have better validation performance with complex validation procedure.
Thank you, we fixed the mistake.

Materials and Methods does not indicate code, libraries, or packages used for model implementations. This is necessary in cases where default hyperparameters are not indicted in text (e.g, in Random Forest what were the max features per tree, etc).
We released the code, hence the Github link is now working. It contains data and implementation details, allowing the community to reproduce our results. We apologize for not having provided such an access before.
